# Crystal structure of 2,5-bis­(4-methyl­pyridin-2-yl)pyrazine chloro­form disolvate^1^

**DOI:** 10.1107/S1600536814009544

**Published:** 2014-08-01

**Authors:** Laurette Schmitt, Helen Stoeckli-Evans

**Affiliations:** a Institute of Physics, University of Neuchâtel, rue Emile-Argand 11, CH-2000 Neuchâtel, Switzerland

**Keywords:** crystal structure, pyrazine, pyridine, bis(2-pyridyl)pyrazine, solvate

## Abstract

The heterocyclic molecule in the title solvate, C_16_H_14_N_4_·2CHCl_3_, possesses inversion symmetry, with the inversion centre situated at the centre of the pyrazine ring. The outer pyridine rings are inclined to the central pyrazine ring by 4.89 (9)°. The compound crystallized as a chloro­form disolvate with the solvent mol­ecules linked to the title mol­ecule by C—H⋯N hydrogen bonds. In the crystal, mol­ecules are further linked by π–π inter­actions involving the pyrazine and pyridine rings of neighbouring mol­ecules [inter-centroid distance = 3.5629 (12) Å; symmetry code: *x*, *y* + 1, *z* + 1].

## Related literature   

The title compound is the 4-methyl­pyridine derivative of the ligand 2,5-bis­(pyridin-2-yl)pyrazine (bppz), see: Neels & Stoeckli-Evans (1993 ▶); Schmitt (2008 ▶). For the synthesis of a number of transition-metal complexes of bppz, especially of copper(II), and for their magnetic properties, see for example: Escuer *et al.* (1993 ▶); Neels *et al.* (1995 ▶); Yuste *et al.* (2009 ▶); Bentama *et al.* (2012 ▶).
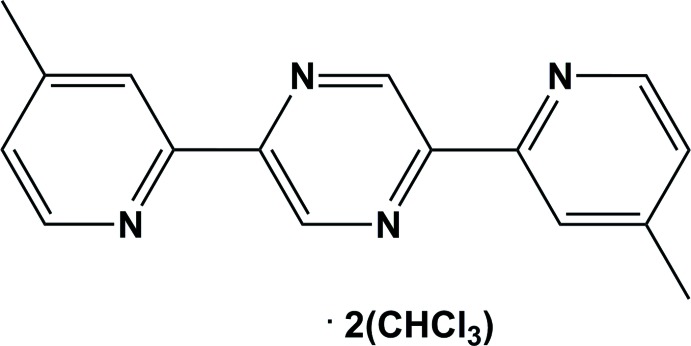


## Experimental   

### Crystal data  

C_16_H_14_N_4_·2CHCl_3_
*M* *_r_* = 501.05
Monoclinic, 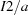

*a* = 16.4498 (17) Å
*b* = 6.0691 (4) Å
*c* = 21.605 (3) Å
β = 93.885 (9)°
*V* = 2152.0 (4) Å^3^
*Z* = 4
Mo *K*α radiation
μ = 0.81 mm^−1^
*T* = 173 K
0.45 × 0.29 × 0.23 mm

### Data collection  

Stoe IPDS 2 diffractometer
Absorption correction: multi-scan (*MULscanABS* in *PLATON*; Spek, 2009 ▶) *T*_min_ = 0.567, *T*_max_ = 1.000
9566 measured reflections
1902 independent reflections
1676 reflections with *I* > 2σ(*I*)
*R*_int_ = 0.066

### Refinement  

*R*[*F* ^2^ > 2σ(*F* ^2^)] = 0.040
*wR*(*F* ^2^) = 0.112
*S* = 1.06
1902 reflections
129 parameters
H-atom parameters constrained
Δρ_max_ = 0.31 e Å^−3^
Δρ_min_ = −0.30 e Å^−3^

### 

Data collection: *X-AREA* (Stoe & Cie, 2009 ▶); cell refinement: *X-AREA*; data reduction: *X-RED32* (Stoe & Cie, 2009 ▶); program(s) used to solve structure: *SHELXS97* (Sheldrick, 2008 ▶); program(s) used to refine structure: *SHELXL2013* (Sheldrick, 2008 ▶); molecular graphics: *Mercury* (Macrae *et al.*, 2008 ▶); software used to prepare material for publication: *SHELXL2013*, *PLATON* (Spek, 2009 ▶) and *publCIF* (Westrip, 2010 ▶).

## Supplementary Material

Crystal structure: contains datablock(s) I, New_Global_Publ_Block. DOI: 10.1107/S1600536814009544/wm0002sup1.cif

Structure factors: contains datablock(s) I. DOI: 10.1107/S1600536814009544/wm0002Isup2.hkl

**Fig. 1.**
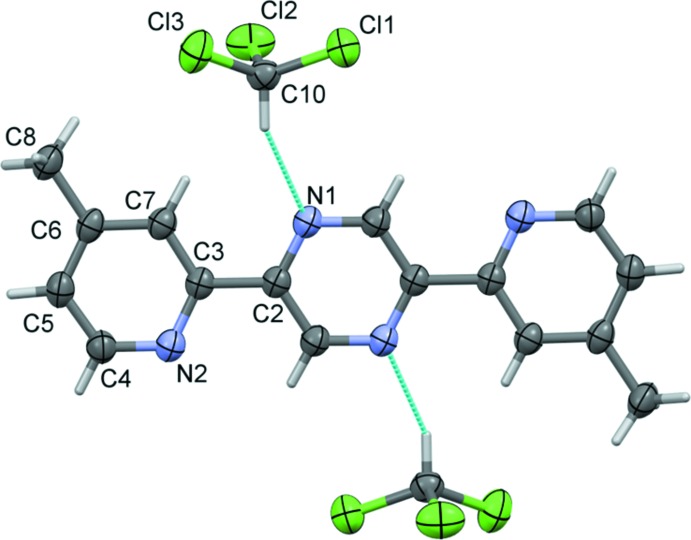
A view of the molecular structure of the title compound, with atom labelling [unlabelled atoms are generated by inversion symmetry (symmetry code: -x + 3/2, -y + 3/2, -z + 1/2)]. Displacement ellipsoids are drawn at the 50% probability level. The C—H···N hydrogen bonds are shown as dashed lines (see Table 1 for details).

. DOI: 10.1107/S1600536814009544/wm0002fig1.tif
A view of the mol­ecular structure of the title compound, with atom labelling [unlabelled atoms are generated by inversion symmetry (symmetry code: −x + 3/2, −y + 3/2, −z + 1/2)]. Displacement ellipsoids are drawn at the 50% probability level. The C—H⋯N hydrogen bonds are shown as dashed lines (see Table 1 for details).

**Fig. 2.**
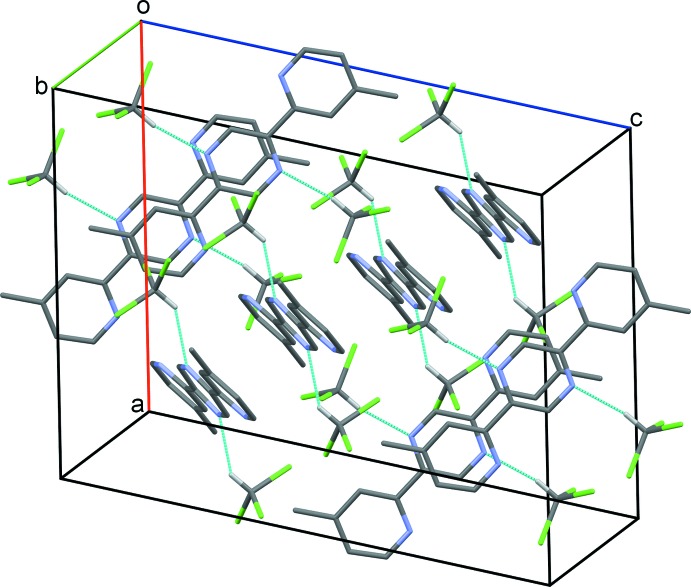
A view of the crystal packing of the title compound, illustrating the overlap of the pyrazine and pyridine rings of neighbouring molecules.

. DOI: 10.1107/S1600536814009544/wm0002fig2.tif
A view of the crystal packing of the title compound, illustrating the overlap of the pyrazine and pyridine rings of neighbouring mol­ecules.

CCDC reference: 1004308

Additional supporting information:  crystallographic information; 3D view; checkCIF report

## Figures and Tables

**Table 1:** Hydrogen-bond geometry (Å, °)

*D*—H⋯*A*	*D*—H	H⋯*A*	*D*⋯*A*	*D*—H⋯*A*
C10—H10⋯N1	1.00	2.50	3.335 (3)	141

## supplementary crystallographic information

### S1.1. Synthesis and crystallization

The title compound was prepared in 3 stages:

Stage 1. Synthesis of methyl-2-pyridine-4-methyl-ketoxime (1): Hydroxyl­amine hydro­chloride (566 mg, 8.14 mmol) in pyridine (5ml) was added to a solution of 2-acetyl-4-methyl­pyrine (1.0 g, 7.40 mmol) in pyridine (22 ml) with vigorous stirring for 3 h at room temperature. The colourless solution was then poured into iced water. The oxime that precipitated was filtered and then dried under vacuum, yielding 1.33 g of compound (1) as a white powder. MS (ESI) calc. for C_8_H_10_N_2_O [M+H]^+^ : 150.18; found 151.1.

Stage 2. Synthesis of methyl-p-tosyl-2-actelypyridine oxime (2): Tosyl chloride (2.40 g, 7.32 mmol) was added to a solution of (1) [1.0 g, 6.6 mmol] in pyridine (10 ml) with stirring for 20 h. The orange solution obtained was poured into iced water. A white precipitate was obtained which was filtered, washed with cold water and dried, yielding compound (2) as a white powder [Yield 74%; 523 mg]. MS (ESI) calc. for C_15_H_16_N_2_O_3_S [M+H]^+^: 304.36; found 305.1.

Stage 3. Synthesis of 2,5-bis­(4-methyl-2-yl)-pyrazine: Potassium tert-butoxide (516 mg, 4.60 mmol) was added in small portions to a solution of (2) [1.0 g, 3.286 mmol] in dry ethanol (15 ml). The mixture was stirred at room temperature for 20 h. The precipitate of potassium p-toluene­sulfonate that formed was filtered off and washed with ether. The filtrate and the washed fractions were collected. This process was repeated until no further precipitation occurred. An aqueous solution of hydrogen peroxide (30%, 4 ml) was then added to the assembled filtrates and washed fractions. When the exothermic reaction had stopped, the mixture was placed in a refrigerator over night yielding the title compound. It was filtered off and washed with water/methanol (1/1, v/v) and dried (yield 5%, 43 mg). MS (ESI) calc. for C_16_H_14_N_4_ [M+H]^+^: 262.31, found 263.13. The colourless rod-like crystals used for X-ray diffraction analysis were obtained by slow evaporation of a solution in chloro­form.

### S1.2. Refinement

The C-bound H atoms were included in calculated positions and treated as riding atoms: C—H = 0.95 - 1.00 Å with *U*_iso_(H) = 1.5*U*_eq_(C-methyl) and = 1.2*U*_eq_(C) for other H atoms.

### Crystal data

**Table:**

C_16_H_14_N_4_·2CHCl_3_	*F*(000) = 1016
*M**_r_* = 501.05	*D*_x_ = 1.546 Mg m^−^^3^
Monoclinic, *I*2/*a*	Mo *K*α radiation, λ = 0.71073 Å
*a* = 16.4498 (17) Å	Cell parameters from 12496 reflections
*b* = 6.0691 (4) Å	θ = 1.5–25.6°
*c* = 21.605 (3) Å	µ = 0.81 mm^−^^1^
β = 93.885 (9)°	*T* = 173 K
*V* = 2152.0 (4) Å^3^	Rod, colourless
*Z* = 4	0.45 × 0.29 × 0.23 mm

### Data collection

**Table:**

Stoe IPDS 2 diffractometer	1902 independent reflections
Radiation source: fine-focus sealed tube	1676 reflections with *I* > 2σ(*I*)
Plane graphite monochromator	*R*_int_ = 0.066
φ + ω scans	θ_max_ = 25.2°, θ_min_ = 1.9°
Absorption correction: multi-scan (*MULscanABS* in *PLATON*; Spek, 2009)	*h* = −19→19
*T*_min_ = 0.567, *T*_max_ = 1.000	*k* = −7→7
9566 measured reflections	*l* = −25→25

### Refinement

**Table:**

Refinement on *F*^2^	Hydrogen site location: inferred from neighbouring sites
Least-squares matrix: full	H-atom parameters constrained
*R*[*F*^2^ > 2σ(*F*^2^)] = 0.040	*w* = 1/[σ^2^(*F*_o_^2^) + (0.0725*P*)^2^ + 0.8241*P*] where *P* = (*F*_o_^2^ + 2*F*_c_^2^)/3
*wR*(*F*^2^) = 0.112	(Δ/σ)_max_ = 0.002
*S* = 1.06	Δρ_max_ = 0.31 e Å^−^^3^
1902 reflections	Δρ_min_ = −0.30 e Å^−^^3^
129 parameters	Extinction correction: *SHELXL2013* (Sheldrick, 2008), Fc^*^=kFc[1+0.001xFc^2^λ^3^/sin(2θ)]^-1/4^
0 restraints	Extinction coefficient: 0.0064 (11)

### Special details

**Table:**

Geometry. All e.s.d.'s (except the e.s.d. in the dihedral angle between two l.s. planes) are estimated using the full covariance matrix. The cell e.s.d.'s are taken into account individually in the estimation of e.s.d.'s in distances, angles and torsion angles; correlations between e.s.d.'s in cell parameters are only used when they are defined by crystal symmetry. An approximate (isotropic) treatment of cell e.s.d.'s is used for estimating e.s.d.'s involving l.s. planes.

### Fractional atomic coordinates and isotropic or equivalent isotropic displacement parameters (Å^2^)

**Table:**

	*x*	*y*	*z*	*U*_iso_*/*U*_eq_	
N1	0.72397 (10)	0.6344 (3)	0.19653 (8)	0.0368 (4)	
N2	0.89303 (10)	0.2947 (3)	0.25318 (8)	0.0394 (4)	
C1	0.68638 (12)	0.8157 (3)	0.21400 (9)	0.0374 (4)	
H1	0.6406	0.8677	0.1890	0.045*	
C2	0.78878 (11)	0.5676 (3)	0.23260 (8)	0.0328 (4)	
C3	0.83238 (11)	0.3674 (3)	0.21341 (9)	0.0328 (4)	
C4	0.93307 (13)	0.1154 (3)	0.23585 (10)	0.0418 (5)	
H4	0.9771	0.0635	0.2627	0.050*	
C5	0.91455 (13)	0.0006 (3)	0.18141 (10)	0.0401 (5)	
H5	0.9446	−0.1274	0.1720	0.048*	
C6	0.85181 (12)	0.0739 (3)	0.14075 (9)	0.0376 (5)	
C7	0.81112 (11)	0.2637 (3)	0.15755 (9)	0.0378 (5)	
H7	0.7685	0.3224	0.1305	0.045*	
C8	0.82828 (15)	−0.0466 (4)	0.08138 (11)	0.0510 (6)	
H8A	0.7910	−0.1672	0.0898	0.077*	
H8B	0.8772	−0.1066	0.0642	0.077*	
H8C	0.8012	0.0556	0.0515	0.077*	
Cl1	0.50499 (4)	0.55130 (10)	0.09961 (3)	0.0569 (2)	
Cl2	0.65296 (4)	0.52518 (12)	0.03495 (3)	0.0617 (3)	
Cl3	0.57200 (4)	0.12697 (10)	0.06982 (3)	0.0582 (2)	
C10	0.59461 (13)	0.4006 (3)	0.09061 (10)	0.0416 (5)	
H10	0.6275	0.3999	0.1312	0.050*	

### Atomic displacement parameters (Å^2^)

**Table:**

	*U*^11^	*U*^22^	*U*^33^	*U*^12^	*U*^13^	*U*^23^
N1	0.0398 (9)	0.0332 (9)	0.0377 (9)	−0.0008 (7)	0.0045 (7)	−0.0041 (7)
N2	0.0429 (9)	0.0351 (9)	0.0405 (9)	0.0005 (7)	0.0053 (7)	−0.0032 (7)
C1	0.0404 (10)	0.0333 (10)	0.0385 (10)	−0.0002 (8)	0.0035 (8)	−0.0027 (8)
C2	0.0356 (10)	0.0294 (10)	0.0340 (10)	−0.0039 (8)	0.0075 (7)	0.0008 (8)
C3	0.0343 (9)	0.0290 (9)	0.0358 (10)	−0.0042 (7)	0.0090 (7)	−0.0001 (7)
C4	0.0467 (11)	0.0374 (11)	0.0417 (11)	0.0052 (9)	0.0054 (8)	−0.0004 (8)
C5	0.0460 (11)	0.0326 (10)	0.0433 (11)	0.0022 (8)	0.0140 (9)	−0.0003 (8)
C6	0.0424 (10)	0.0343 (10)	0.0377 (10)	−0.0038 (8)	0.0136 (8)	−0.0047 (8)
C7	0.0388 (10)	0.0381 (11)	0.0372 (10)	−0.0011 (8)	0.0077 (8)	−0.0025 (8)
C8	0.0578 (13)	0.0499 (13)	0.0460 (12)	0.0046 (10)	0.0076 (10)	−0.0152 (10)
Cl1	0.0557 (4)	0.0538 (4)	0.0613 (4)	0.0130 (3)	0.0040 (3)	−0.0024 (3)
Cl2	0.0589 (4)	0.0759 (5)	0.0507 (4)	−0.0204 (3)	0.0069 (3)	0.0140 (3)
Cl3	0.0758 (5)	0.0417 (4)	0.0594 (4)	−0.0037 (3)	0.0207 (3)	−0.0062 (2)
C10	0.0449 (11)	0.0426 (11)	0.0376 (11)	−0.0012 (9)	0.0041 (8)	0.0024 (9)

### Geometric parameters (Å, º)

**Table:**

N1—C1	1.329 (3)	C5—H5	0.9500
N1—C2	1.340 (3)	C6—C7	1.393 (3)
N2—C4	1.339 (3)	C6—C8	1.504 (3)
N2—C3	1.346 (3)	C7—H7	0.9500
C1—C2^i^	1.391 (3)	C8—H8A	0.9800
C1—H1	0.9500	C8—H8B	0.9800
C2—C1^i^	1.391 (3)	C8—H8C	0.9800
C2—C3	1.484 (3)	Cl1—C10	1.757 (2)
C3—C7	1.385 (3)	Cl2—C10	1.760 (2)
C4—C5	1.383 (3)	Cl3—C10	1.753 (2)
C4—H4	0.9500	C10—H10	1.0000
C5—C6	1.383 (3)		
			
C1—N1—C2	116.90 (17)	C5—C6—C8	121.56 (19)
C4—N2—C3	116.44 (17)	C7—C6—C8	121.58 (19)
N1—C1—C2^i^	122.74 (18)	C3—C7—C6	120.36 (18)
N1—C1—H1	118.6	C3—C7—H7	119.8
C2^i^—C1—H1	118.6	C6—C7—H7	119.8
N1—C2—C1^i^	120.36 (17)	C6—C8—H8A	109.5
N1—C2—C3	117.77 (16)	C6—C8—H8B	109.5
C1^i^—C2—C3	121.87 (17)	H8A—C8—H8B	109.5
N2—C3—C7	122.68 (17)	C6—C8—H8C	109.5
N2—C3—C2	116.19 (16)	H8A—C8—H8C	109.5
C7—C3—C2	121.13 (17)	H8B—C8—H8C	109.5
N2—C4—C5	124.3 (2)	Cl3—C10—Cl1	110.88 (12)
N2—C4—H4	117.9	Cl3—C10—Cl2	110.37 (12)
C5—C4—H4	117.9	Cl1—C10—Cl2	110.64 (12)
C6—C5—C4	119.35 (19)	Cl3—C10—H10	108.3
C6—C5—H5	120.3	Cl1—C10—H10	108.3
C4—C5—H5	120.3	Cl2—C10—H10	108.3
C5—C6—C7	116.86 (18)		
			
C2—N1—C1—C2^i^	−0.8 (3)	C3—N2—C4—C5	−1.7 (3)
C1—N1—C2—C1^i^	0.7 (3)	N2—C4—C5—C6	1.2 (3)
C1—N1—C2—C3	−179.12 (16)	C4—C5—C6—C7	0.6 (3)
C4—N2—C3—C7	0.5 (3)	C4—C5—C6—C8	−179.05 (19)
C4—N2—C3—C2	−179.40 (16)	N2—C3—C7—C6	1.2 (3)
N1—C2—C3—N2	−174.82 (17)	C2—C3—C7—C6	−178.94 (16)
C1^i^—C2—C3—N2	5.3 (3)	C5—C6—C7—C3	−1.7 (3)
N1—C2—C3—C7	5.3 (3)	C8—C6—C7—C3	177.96 (19)
C1^i^—C2—C3—C7	−174.56 (18)		

Symmetry code: (i) −*x*+3/2, −*y*+3/2, −*z*+1/2.

### Hydrogen-bond geometry (Å, º)

**Table:**

*D*—H···*A*	*D*—H	H···*A*	*D*···*A*	*D*—H···*A*
C10—H10···N1	1.00	2.50	3.335 (3)	141
