# Supplementary figures and images for: Crystal structure of 2,5-bis­(4-methyl­pyridin-2-yl)pyrazine chloro­form disolvate
Source: Acta Crystallogr Sect E Struct Rep Online. 2014 Aug 1;70(Pt 9):o893–4. doi: 10.1107/S1600536814009544 (PMC4186168; doi:10.1107/S1600536814009544)

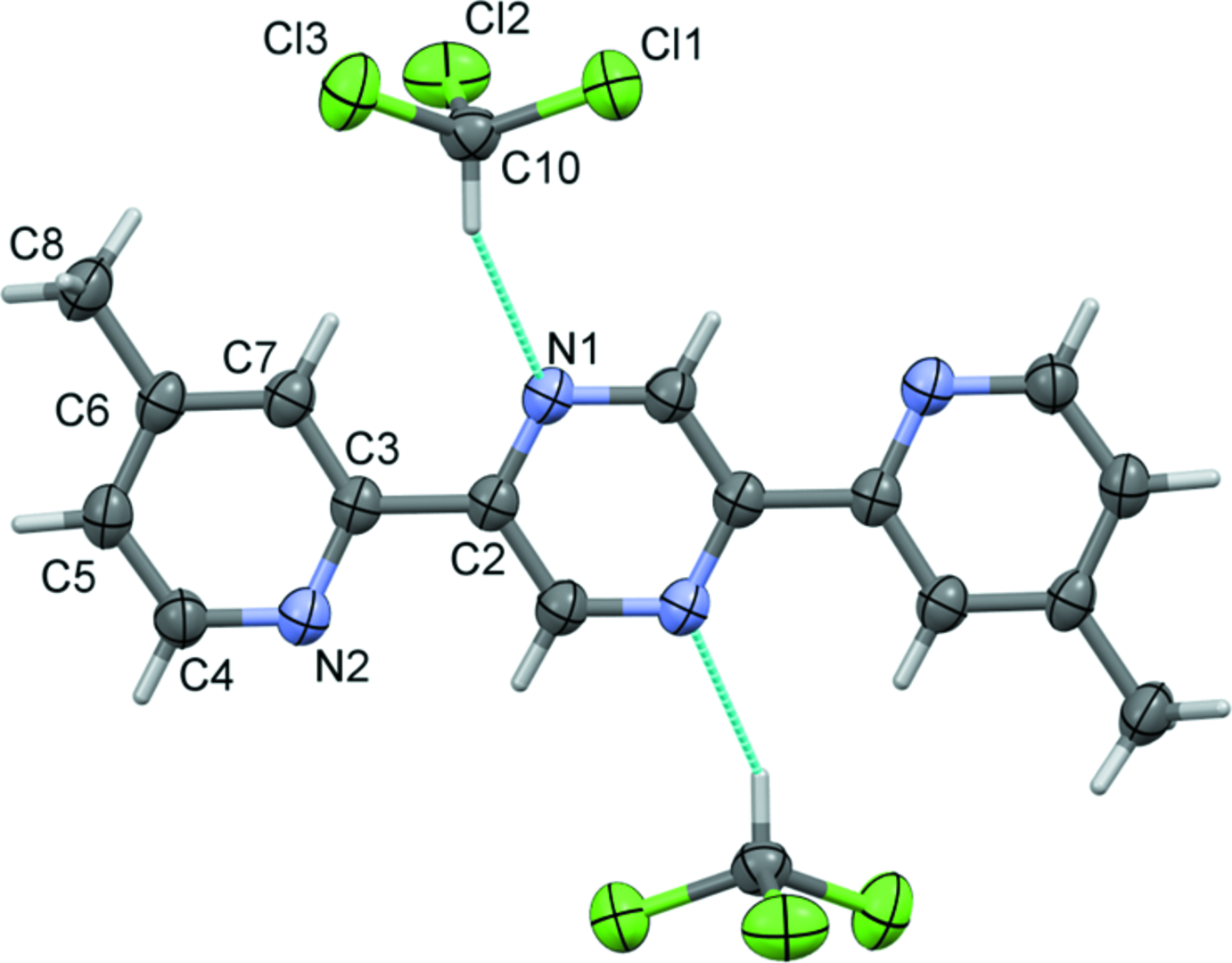

Supplement: Supplementary file 3 [file e-70-0o893-fig1.tif]

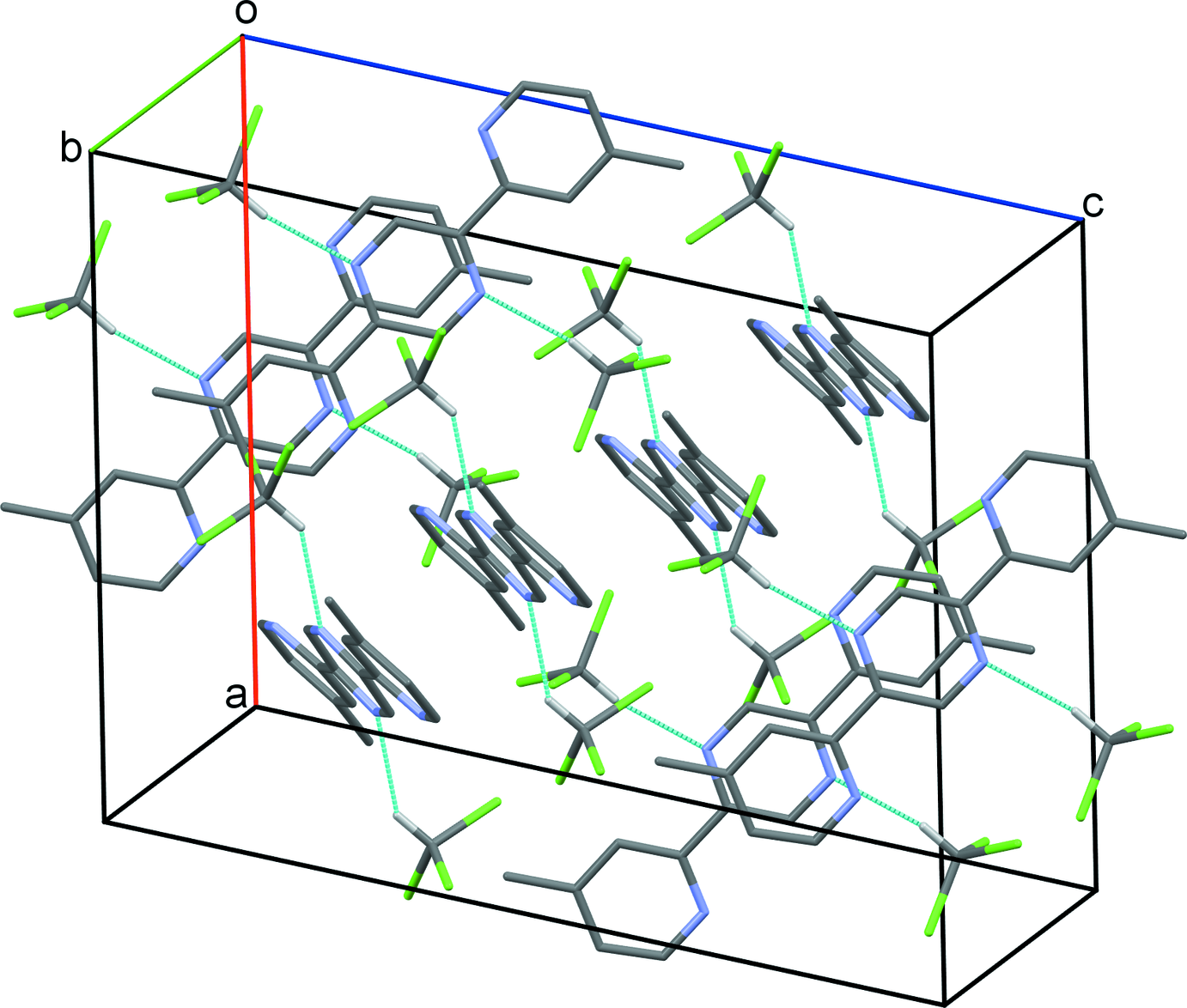

Supplement: Supplementary file 4 [file e-70-0o893-fig2.tif]
